# Septic Pulmonary Embolism Requiring Critical Care: Clinicoradiological Spectrum, Causative Pathogens and Outcomes

**DOI:** 10.6061/clinics/2016(10)02

**Published:** 2016-10

**Authors:** Deng-Wei Chou, Shu-Ling Wu, Kuo-Mou Chung, Shu-Chen Han, Bruno Man-Hon Cheung

**Affiliations:** I Department of Critical Care Medicine, Tainan Municipal Hospital, Tainan/Taiwan; II Department of Nursing, Chung-Hwa University of Medical Technology, Tainan/Taiwan; III Department of Long Term Care, Chung-Hwa University of Medical Technology, Tainan/Taiwan; IV Department of Internal Medicine, Tainan Municipal Hospital, Tainan/Taiwan; V Department of Radiology, Tainan Municipal Hospital, Tainan/Taiwan

**Keywords:** Septic Pulmonary Embolism, Intensive Care Unit, Pneumonia, Liver Abscess

## Abstract

**OBJECTIVES:**

Septic pulmonary embolism is an uncommon but life-threatening disorder. However, data on patients with septic pulmonary embolism who require critical care have not been well reported. This study elucidated the clinicoradiological spectrum, causative pathogens and outcomes of septic pulmonary embolism in patients requiring critical care.

**METHODS:**

The electronic medical records of 20 patients with septic pulmonary embolism who required intensive care unit admission between January 2005 and December 2013 were reviewed.

**RESULTS:**

Multiple organ dysfunction syndrome developed in 85% of the patients, and acute respiratory failure was the most common organ failure (75%). The most common computed tomographic findings included a feeding vessel sign (90%), peripheral nodules without cavities (80%) or with cavities (65%), and peripheral wedge-shaped opacities (75%). The most common primary source of infection was liver abscess (40%), followed by pneumonia (25%). The two most frequent causative pathogens were *Klebsiella pneumoniae* (50%) and *Staphylococcus aureus* (35%). Compared with survivors, nonsurvivors had significantly higher serum creatinine, arterial partial pressure of carbon dioxide, and Acute Physiology and Chronic Health Evaluation II and Sequential Organ Failure Assessment scores, and they were significantly more likely to have acute kidney injury, disseminated intravascular coagulation and lung abscesses. The in-hospital mortality rate was 30%. Pneumonia was the most common cause of death, followed by liver abscess.

**CONCLUSIONS:**

Patients with septic pulmonary embolism who require critical care, especially those with pneumonia and liver abscess, are associated with high mortality. Early diagnosis, appropriate antibiotic therapy, surgical intervention and respiratory support are essential.

## INTRODUCTION

Septic pulmonary embolism (SPE) is an uncommon disorder in which a microorganism-containing thrombus causes an inflammatory reaction and a mechanical obstruction in the pulmonary vasculature 1,2. Typical findings of SPE on computed tomography (CT) include peripheral nodules with or without cavitations, a feeding vessel sign, wedge-shaped peripheral lesions abutting the pleura and abscesses 2–5. Most patients with SPE are diagnosed on the basis of CT findings and the presence of a primary source of infection 2–4 because histopathological confirmation is typically unfeasible in clinical practice 2. The clinical presentations of SPE range from insidious illness with mild respiratory symptoms to respiratory failure and septic shock 6. Despite its life-threatening nature, data on patients with SPE who require critical care have not been well reported. Therefore, the aim of this study was to elucidate the clinicoradiological spectrum, causative pathogens, and outcomes of SPE for patients requiring intensive care unit (ICU) admission.

## MATERIALS AND METHODS

### Patient selection

This study was conducted in the ICU (mixed medical-surgical 40-bed unit with approximately 2,300 annual admissions) of Tainan Municipal Hospital, a referral teaching hospital in Southern Taiwan. We performed a retrospective computer-aided search to identify the electronic medical records of patients consecutively admitted to the ICU with a diagnosis of SPE between January 2005 and December 2013. Our inclusion criteria for SPE diagnosis were adapted from Cook et al. 2: a CT scan showing multiple nodular opacities or multifocal lung infiltrates compatible with septic embolism to the lung; the presence of a primary source of infection as a potential embolic source; and clinical and radiographic improvement after antibiotic therapy. Patients with a diagnosis of lung tumor, tuberculosis, or possible lung metastasis were excluded from the study. In total, 20 (12 male and 8 female) patients met the study criteria.

### Data collection

The following data for the study patients were collected: age, gender, underlying conditions, biochemistry results, causative pathogens, Acute Physiology and Chronic Health Evaluation II (APACHE-II) score at ICU admission, Sequential Organ Failure Assessment (SOFA) score at ICU admission, clinical complications, length of stay in ICU and outcome.

### CT examinations

During the 9-year study period, CT scans were obtained using two multidetector CT scanners, namely, a GE LightSpeed VCT 64-slice CT scanner (GE Healthcare, Milwaukee, USA) and a GE BrightSpeed Elite Select 16-slice CT scanner (GE Healthcare, Milwaukee, USA). The CT scanning parameters were as follows: 120 kV, 100–320 mA, 0.8-second rotation time, and 5-mm collimation (a feeding vessel sign was obtained with 1.25-mm collimation on a lung window). The images were reviewed at a lung-window setting level of -700 Hounsfield units (HU) with a width of 1500 HU and a mediastinum-window setting level of 40 HU with a width of 350 HU. Thoracic CT examinations were performed within 7 days of admission.

### Image interpretation

Two chest physicians (Chou DW and Chung KM with 17 and 22 y of experience in chest CT image interpretation, respectively) independently evaluated the thoracic CT images and follow-up chest radiographs from the 20 patients. The images were assessed for the following radiological patterns: a feeding vessel sign, peripheral wedge-shaped opacities, nodules with or without cavities, patchy ground-glass opacities, focal consolidations, lung abscesses and pleural effusions. Discrepancies were resolved by an experienced chest radiologist (Han SC, who has 20 y of experience in chest CT image interpretation).

### Statistical analysis

Data were analyzed using SPSS Version 16.0 (SPSS Inc., Chicago, IL, USA). Categorical and continuous variables of survivors and non-survivors were compared using Fisher's exact test and the Mann-Whitney U test, respectively. All data are expressed as the mean (±SD). For all analyses, *p*<0.05 was considered statistically significant.

### Definitions

APACHE-II and SOFA scores at ICU admission were calculated as described in the literature 7,8. Acute respiratory failure was defined as an arterial partial pressure of oxygen <60 mmHg or an arterial partial pressure of carbon dioxide (PaCO_2_) >50 mmHg to produce respiratory acidosis (pH <7.35) while breathing air at normal atmospheric pressure. The diagnostic criterion for acute kidney injury (AKI) was an increase in serum creatinine by at least 0.3 mg/dL within 48 hours 9. The Berlin definition of acute respiratory distress syndrome (ARDS) was employed 10. Disseminated intravascular coagulation (DIC) was diagnosed using a scoring system proposed by the International Society on Thrombosis and Haemostasis 11. Ground-glass attenuation (GGA) was defined as a hazy increase in attenuation without obscuration of vascular markings, and focal consolidation was defined as a localized increase in lung attenuation with obscuration of vascular markings 12.

### Ethics

We could not obtain informed consent from the patients due to the use of a retrospective study design. Therefore, we posted an announcement about the study. This study does not present data that could be used to identify the patients and was approved by the Institutional Review Board (SCMH IRB: 1011202) of Tainan Municipal Hospital.

## RESULTS

The study included 12 male and 8 female patients with a mean age of 56.5±13.0 years (range, 29–80 y). All 20 patients were admitted through the emergency department, and 15 (75%) were directly admitted to the ICU. The diagnosis of SPE was made on the day of admission for 10 (50%) patients and within 7 days of admission for the remaining 10 (50%) patients. Table 1 presents a comparison of the clinicoradiological characteristics of the survivors and nonsurvivors. Diabetes mellitus was the major underlying condition (*n*=13, 65%). Six patients (30%) died during hospitalization, and fourteen patients (70%) recovered from their illness. The survivors and nonsurvivors did not significantly differ in demographic data, pathogens, or length of stay in the ICU. Regarding underlying conditions, a history of hypertension was significantly more common (50% *vs.* 7%, *p*=0.028) in the nonsurvivors; however, no significant difference was observed in the incidence of diabetes mellitus, cerebrovascular disease, excessive alcoholic consumption or end-stage renal disease. Regarding biochemistry data, the nonsurvivors had significantly higher serum creatinine (2.93±2.4 *vs.* 1.49±0.5, *p*=0.039) and PaCO_2_ (42.7±13.0 *vs.* 29.7±9.3, *p*=0.021) levels than the survivors. However, the nonsurvivors had significantly lower arterial pH (7.22±0.2 *vs.* 7.39±0.1, *p*=0.01). Serum C-reactive protein and sodium levels were non-significantly higher in the nonsurvivors compared to the survivors (*p*=0.053 and 0.056, respectively). No differences were seen in white blood cell count, platelets, albumin, blood urea nitrogen, potassium, arterial HCO_3_^-^, or glycosylated hemoglobin. At ICU admission, the nonsurvivors had significantly higher mean APACHE II (31±6.0 *vs.* 18±6.0, *p*<0.001) and mean SOFA scores (12±3.4 *vs.* 8±2.6, *p*=0.01) compared to the survivors. The most common complication was acute respiratory failure (*n*=15, 75%), followed by septic shock (*n*=12, 60%), AKI (*n*=5, 25%), acute respiratory distress syndrome (*n*=3, 15%) and DIC (*n*=2, 10%). Compared with the survivors, the incidences of AKI (67% *vs.* 7%, *p*=0.005) and DIC (33% *vs.* 0, *p*=0.023) were significantly higher in the nonsurvivors. The mean length of stay in the ICU was 12.6±8.9 days. Six patients died of multiple organ dysfunction syndrome (MODS) during hospitalization (for an in-hospital mortality or 30%). The post-hospital discharge follow-up duration for the 14 survivors ranged from 4 to 48 months; none of the survivors had SPE-related complications.

More than two imaging findings were obtained for each patient. Sixteen (80%) patients exhibited lesions in the bilateral lungs. The CT findings included a feeding vessel sign (90%), nodules without cavities (80%), peripheral wedge-shaped opacities (75%), nodules with cavities (65%), pleural effusions (65%), focal consolidations (40%), lung abscesses (30%), and patchy ground-glass opacities (20%). Compared with the survivors, the nonsurvivors were significantly more likely to have lung abscesses (67% *vs.* 14%, *p*=0.019). Follow-up chest radiography for the 14 survivors revealed that ill-defined infiltrates and nodular opacities were resolved within 7–14 days and 14–60 days, respectively.

**Figure 1:**
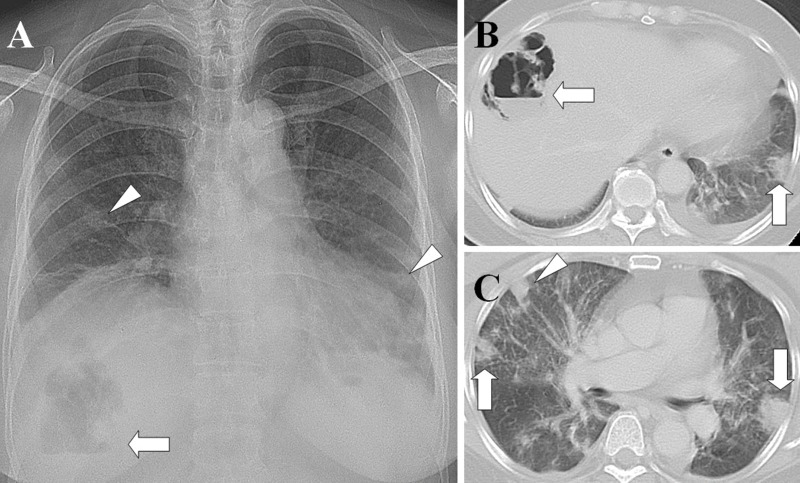
Liver abscess with septic pulmonary emboli. (A) A chest radiograph shows an air-fluid level in the right upper abdomen (arrow) and multiple nodular opacities in the bilateral lungs (arrowheads). (B) A CT scan shows a gas-forming liver abscess (arrow) and a peripheral wedge-shaped opacity abutting the adjacent pleura (arrow). (C) A lung window of a cross-sectional CT scan shows two peripheral wedge-shaped opacities abutting the adjacent pleura (arrows) and a peripheral nodule with a feeding vessel (arrowhead). The patient was a 61-year-old diabetic woman whose blood and aspirate abscess cultures were positive for *Klebsiella pneumoniae*.

**Figure 2:**
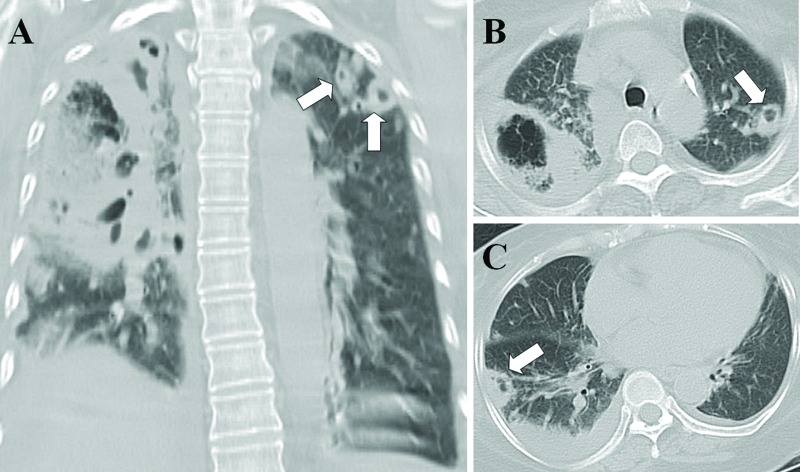
Necrotizing pneumonia with septic pulmonary emboli. (A) A lung window of a coronal-sectional CT scan shows necrotizing pneumonia in the right lung. Multiple different sizes of nodules with cavities in the left upper lobe (arrows), suggestive of septic pulmonary emboli, are observed. (B) A lung window of a cross-sectional CT scan shows necrotizing pneumonia in the right upper lobe and a cavitary nodule in the left upper lobe (arrows). (C) A peripheral wedge-shaped opacity abutting the adjacent pleura in the right lower lobe (arrow) and pleural effusion are seen. The patient was a 62-year-old diabetic woman whose blood and sputum cultures were positive for methicillin-resistant *Staphylococcus aureus*.

**Figure 3:**
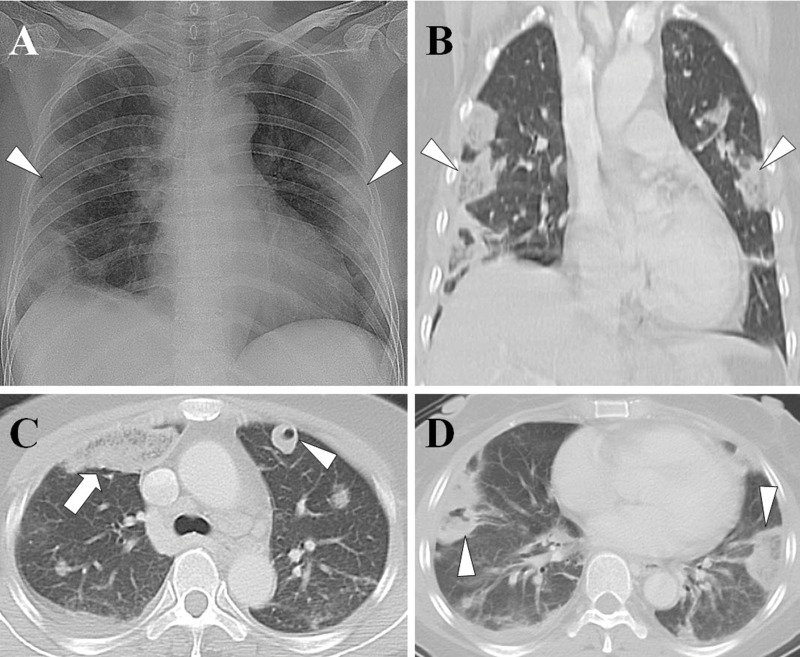
Tricuspid valve infective endocarditis with septic pulmonary emboli. (A) A chest radiograph shows multiple peripheral patchy opacities in the bilateral lungs (arrowheads). (B) A lung window of a coronal CT scan shows multiple peripheral wedge-shaped opacities (arrowheads). (C) A lung window of a cross-sectional CT scan shows a ground-glass opacity in the right upper lobe (arrow) and a nodule with cavity in the left upper lobe (arrowhead). (D) A peripheral wedge-shaped opacity with central necrosis in the right lower lobe (arrowhead) and a peripheral wedge-shaped opacity without central necrosis in the left lower lobe (arrowhead). The patient was a 50-year-old woman whose blood cultures were positive for methicillin-susceptible *Staphylococcus aureus*.

**Figure 4:**
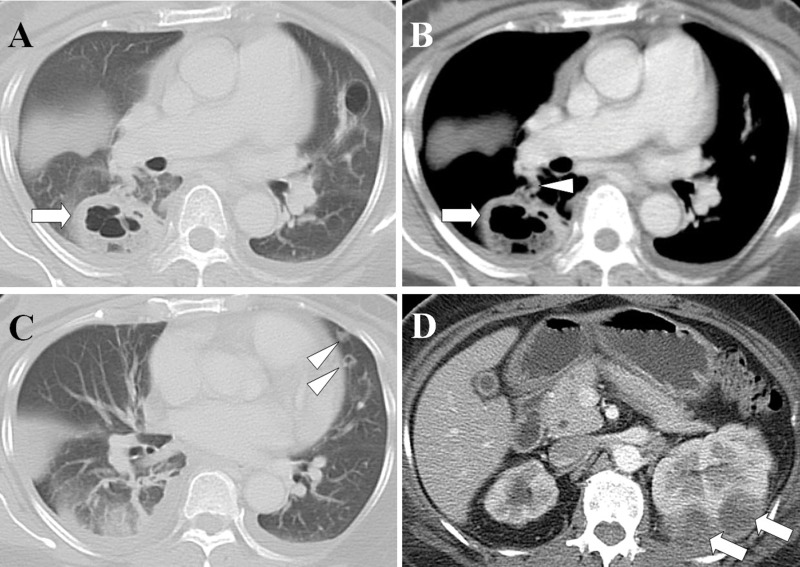
Renal abscesses with septic pulmonary emboli. (A) A lung window of a cross-sectional CT scan shows a lung abscess with a diameter of 4.5 cm in the right lower lobe (arrow). (B) A contrast-enhanced CT scan (mediastinum window) in the same image plane shows a lung abscess (arrow) with a feeding vessel sign (arrowhead). (C) A lung window of a cross-sectional CT scan shows two nodules with cavities in the left upper lobe (arrowheads). (D) An abdominal CT scan shows left renal abscesses (arrows). The patient was a 52-year-old woman whose blood cultures were positive for *Escherichia coli*.

Table 2 shows the causative pathogens, primary sources of infection, and surgical procedures for the 20 patients. All 20 patients had positive blood cultures. The most common causative pathogen was *Klebsiella pneumoniae* (*n*=10, 50%), followed by *Staphylococcus aureus* (*n*=7, 35%), *Pseudomonas aeruginosa* (*n*=1, 5%), *Escherichia coli* (*n*=1, 5%), and *Salmonella* group B (*n*=1, 5%). The most common primary source of infection was liver abscess (Figure 1) (*n*=8, 40%), followed by pneumonia (Figure 2) (*n*=5, 25%), tricuspid valve infective endocarditis (Figure 3) (*n*=3, 15%), renal abscess (Figure 4) (*n*=2, 10%), deep neck infection (*n*=1, 5%) and soft tissue abscess (*n*=1, 5%). Three, two and one of the nonsurvivors had pneumonia, liver abscess and deep neck infection, respectively. Among the ten patients with *K. pneumoniae* as the causative pathogen, eight, one and one patient had liver abscess, deep neck infection and renal abscess, respectively. Among the seven patients with *S. aureus* as the causative pathogen, four had pneumonia and three had tricuspid valve infective endocarditis. Antibiotic therapy was initiated in all patients on the day of admission and was administered for 3–6 weeks in the survivors. Transthoracic (*n*=7) and transesophageal (*n*=4) echocardiography were performed in the seven patients whose blood cultures were positive for *S. aureus*. Tricuspid valve vegetation was proved in three patients. Overall, six patients underwent percutaneous pigtail catheter drainage of liver abscess, three underwent video-assisted thoracostomy surgery with decortications for loculated pleural effusion, two underwent tube thoracostomy for massive pleural effusions, two underwent percutaneous pigtail catheter drainage of renal abscess, one underwent tube thoracostomy for empyema and one underwent tricuspid valve replacement.

## DISCUSSION

During the study period, 32 patients were diagnosed with SPE in our institution. Twenty (63%) of these patients required ICU admission; all were admitted through the emergency department. Fifteen (75%) patients were directly admitted to the ICU, indicating that SPEs can occur fulminantly and seriously. MODS developed in 85% (17 of 20) of the patients, and acute respiratory failure was the most common organ failure (75%). Nonsurvivors had significantly higher serum creatinine and PaCO_2_ levels as well as significantly higher APACHE II and SOFA scores. AKI, DIC, and lung abscess were significantly more prevalent in non-survivors.

Historically, the most common primary source of infection has been tricuspid valve infective endocarditis secondary to intravenous drug abuse 13. However, the epidemiology of SPE has changed over the past 30 years 2. In the current study, liver abscess, pneumonia, and tricuspid valve infective endocarditis were the major primary sources of SPE, and *K. pneumoniae* liver abscess (KPLA) was the most common primary source of SPE (40%, 8 of 20). Taiwan has one of the highest prevalences of KPLA worldwide 14, and the lung is among the most common sites of metastatic infection 15. All our *K. pneumoniae* bacteremic isolates expressed the hypermucoviscous phenotype, which has been associated with the development of a distinctive type of invasive liver abscess syndrome 16. The hypermucoviscous phenotype of the capsular serotype K1 is a major virulence factor of *K. pneumoniae* that can cause primary liver abscess and catastrophic septic metastatic complications and is an emerging pathogen 17,18. Physicians should therefore initiate aggressive investigations for invasive liver abscess syndrome when the hypermucoviscous phenotype is identified. All eight patients with KPLA were diabetic and 90% of them exhibited a glycosylated hemoglobin value exceeding 10%, indicating that poor glycemic control is highly associated with SPE. Strict glycemic control can prevent the development of metastatic complications 19.

In 2007, the first two cases of pneumonia as a primary source of SPE, diagnosed using chest images, were reported 20. However, no related case reports have since been published, possibly because of the difficulty of distinguishing SPE from pneumonia through imaging. However, in another study based on postmortem examinations, pneumonia was the most common cause of SPE (34%, 84 of 247) 21. Therefore, the number of patients with pneumonia plus SPE may have been underestimated.

Morikawa et al. reported CT findings, including GGA, centrilobular nodules, consolidations, reticular opacities, nodules, and pleural effusion, from 68 patients with methicillin-resistant *S. aureus* (MRSA) and 83 patients with methicillin-susceptible *S. aureus* (MSSA) pneumonia 22. In addition, Nguyen et al. reported CT findings, including consolidations, multiple nodules and GGA, from nine patients with community-acquired MRSA pneumonia 23. SPE can present as consolidations, multiple nodules with cavities and GGA in CT images 6, all of which are present in pneumonia, thus making distinguishing SPE from pneumonia difficult. In the present study, CT examinations showed multiple different sizes of nodules with cavities, as shown in Figure 2, which is suggestive of SPE instead of pneumonia.

The highest mortality (60%, 3 of 5) occurred in patients with pneumonia as a primary source of SPE. Although *S. aureus* constituted only approximately 3% of community-acquired pneumonia (CAP) 12 cases, community-acquired necrotizing pneumonia caused by *S. aureus* can cause a high mortality rate of approximately 40%–50% 24,25. CAP caused by *P. aeruginosa* is very rare but has been associated with a high mortality rate of approximately 30% 26. Furthermore, in patients with MSSA and *P. aeruginosa* CAP complicated with bacteremia, the in-hospital mortality rates at day 30 can be as high as 61% and 65%, respectively 27. Because of the high mortality associated with bacteremic CAP complicated with SPE, high clinical suspicion is probably the most crucial factor in obtaining an early and rapid diagnosis, especially for MRSA CAP 28. Some strains of *S. aureus* carrying the Panton-Valentine leukocidin gene can cause rapidly progressive and highly lethal necrotizing pneumonia 29. Early and rapid diagnosis using aggressive diagnostic measures and rapid institution of effective therapy are essential for the treatment of pneumonia with SPE.

In the present study, *S. aureus* was the major causative pathogen of tricuspid valve infective endocarditis and pneumonia. However, all the patients with tricuspid valve infective endocarditis recovered from the illness, in contrast to the patients with pneumonia. Among the three patients with tricuspid valve infective endocarditis complicated with SPE, one underwent valve replacement because of recurrent sepsis despite appropriate antibiotic therapy, one underwent thoracotomy because of empyema complicated by SPE, and one received antibiotic therapy only. Surgical interventions, such as valve replacement and thoracotomy, can reduce the mortality rate of tricuspid valve infective endocarditis complicated with SPE.

Only three cases of renal abscess as a primary source of SPE have been reported 30–32. In the present study, we reported two cases of renal abscess as a primary source of SPE. The causative pathogens were *K. pneumoniae* and *E. coli*. Early treatment of the complications of SPE, including lung abscess and empyema, is imperative. Adequate antibiotic therapy, early treatment of empyema, and renal abscess drainage can reduce morbidity and mortality in patients with renal abscess with SPE.

Two limitations were encountered while conducting this study. First, data were collected retrospectively from a single institution, which may have resulted in a selection bias. Second, we could not identify significant predictors of mortality because of the relatively small patient sample. Despite these shortcomings, our study provides critical information on patients with SPE who require critical care.

The main findings of this study are summarized as follows. First, patients with SPE who require critical care, especially those with pneumonia and liver abscess, are often associated with high mortality. Second, the most common primary source of infection is liver abscess, followed by pneumonia and tricuspid valve infective endocarditis. Therefore, when patients with liver abscess, pneumonia, or tricuspid valve infective endocarditis require critical care, physicians should carefully examine the possibility of SPEs. Third, early diagnosis, appropriate antibiotic therapy, surgical intervention and respiratory support are essential for the treatment of patients with SPE who require critical care.

## AUTHOR CONTRIBUTIONS

Chou DW designed the study, interpreted the data and wrote the manuscript. Wu SL collected and analyzed the data. Chou DW, Chung KM and Han SC evaluated the chest radiographs and CT scans. Cheung BM interpreted the microbiological findings.

## Figures and Tables

**Table 1:** Comparison of clinicoradiological characteristics between survivors and nonsurvivors.

Variables	Total *N* =20 (%)	Survivors *N* =14 (%)	Nonsurvivors *N* =6 (%)	*p*
Age (yr)	56.5±13.0	55.7±11.6	58.2±16.8	NS
Gender (M/F)	12/8	7/7	5/1	NS
Direct admission to the intensive care unit	15 (75)	10 (71)	5 (83)	NS
Underlying conditions				
Diabetes mellitus	13 (65)	9 (64)	4 (67)	NS
Hypertension	4 (20)	1 (7)	3 (50)	0.028
Cerebrovascular disease	4 (20)	2 (14)	2 (33)	NS
Excessive alcoholic consumption	2 (10)	1 (7)	1 (17)	NS
End stage renal disease	1 (5)	0	1 (17)	NS
Biochemistry data				
White blood cell count (10^9^/L)	19.4±11.5	22.4±11.4	12.5±8.8	NS
Platelet (10^9^/L)	106.1±108.6	96.5±114.9	128.3±98.3	NS
C-reactive protein (mg/dL)	25.5±9.7	23.4±8.8	37.5±3.1	NS
Albumin (g/dL)	2.5±0.5	2.5±0.4	2.4±0.7	NS
Blood urea nitrogen (mg/dL)	46.9±25.8	40.7±19.7	61.3±34.1	NS
Creatinine (mg/dL)	1.92±1.5	1.49±0.5	2.93±2.4	0.039
Sodium (mEq/L)	135.1±11.0	132.0±8.8	142.2±13.2	NS
Potassium (mEq/L)	3.9±1.1	3.78±0.9	4.16±1.4	NS
Arterial blood gas				
pH	7.34±0.13	7.39±0.1	7.22±0.2	0.01
PaCO_2_ (mmHg)	33.6±11.9	29.7±9.3	42.7±13.0	0.021
HCO_3_^-^ (mmol/L)	17.9±4.6	18.1±3.6	17.6±6.7	NS
Glycosylated hemoglobin (%)	12.3±3.0	12.5±3.6	11.7±0.7	NS
Pathogens				
Gram-negative infection	13 (65)	9 (64)	4 (67)	NS
Gram-positive infection	7 (35)	5 (36)	2 (33)	NS
Scoring systems at ICU admission				
APACHE II score	22±8.6	18±6.0	31±6.0	<0.001
SOFA score	9±3.3	8±2.6	12±3.4	0.01
Complications				
Acute respiratory failure	15 (75)	10 (71)	5 (83)	NS
Septic shock	12 (60)	7 (50)	5 (83)	NS
Acute kidney injury	5 (25)	1 (7)	4 (67)	0.005
Acute respiratory distress syndrome	3 (15)	1 (7)	2 (33)	NS
Disseminated intravascular coagulopathy	2 (10)	0	2 (33)	0.023
Length of stay in ICU (d)	12.6±8.9	10.7±3.7	17.0±15.1	NS
Computed tomographic findings				
A feeding vessel sign	18 (90)	12 (86)	6 (100)	NS
Nodule without cavity	16 (80)	11 (79)	5 (83)	NS
Peripheral wedge-shaped opacity	15 (75)	9 (64)	6 (100)	NS
Nodule with cavity	13 (65)	9 (64)	4 (67)	NS
Pleural effusion	13 (65)	10 (71)	3 (50)	NS
Focal consolidation	8 (40)	5 (36)	3 (50)	NS
Lung abscess	6 (30)	2 (14)	4 (67)	0.019
Patchy ground-glass attenuation	4 (20)	2 (14)	2 (33)	NS
Bilateral lesions	16 (80)	11 (79)	5 (83)	NS

APACHE: Acute Physiology and Chronic Health Evaluation; ICU: Intensive care unit; NS: Not significant; SOFA: Sequential Organ Failure Assessment

**Table 2:** Causative pathogens, primary sources of infection, and surgical procedures for the 20 patients

No.	Pathogen	Primary source of infection	Culture site	Procedure	Outcome
1	Methicillin-susceptible *S. aureus*	Tricuspid valve infective endocarditis	Blood	TVR	Survivor
2	Methicillin-susceptible *S. aureus*	Tricuspid valve infective endocarditis	Blood	VATS	Survivor
3	Methicillin-susceptible *S. aureus*	Tricuspid valve infective endocarditis	Blood		Survivor
4	Methicillin-resistant *S. aureus*	Pneumonia	Blood, sputum		Nonsurvivor
5	Methicillin-susceptible *S. aureus*	Pneumonia	Blood, sputum		Nonsurvivor
6	Methicillin-susceptible *S. aureus*	Pneumonia	Blood, sputum	VATS	Survivor
7	Methicillin-susceptible *S. aureus*	Pneumonia	Blood, sputum		Survivor
8	*K. pneumoniae*	Liver abscess	Blood, abscess, cerebrospinal fluid	PCD	Nonsurvivor
9	*K. pneumoniae*	Liver abscess	Blood, abscess	PCD	Nonsurvivor
10	*K. pneumoniae*	Liver abscess	Blood, abscess	PCD, tube thoracostomy	Survivor
11	*K. pneumoniae*	Liver abscess	Blood, abscess	PCD, tube thoracostomy	Survivor
12	*K. pneumoniae*	Liver abscess	Blood, abscess	PCD, VATS	Survivor
13	*K. pneumoniae*	Liver abscess	Blood, abscess	PCD	Survivor
14	*K. pneumoniae*	Liver abscess	Blood		Survivor
15	*K. pneumoniae*	Liver abscess	Blood		Survivor
16	*K. pneumoniae*	Deep neck infection	Blood		Nonsurvivor
17	*K. pneumoniae*	Renal abscess	Blood, abscess, urine	VATS, PCD	Survivor
18	*P. aeruginosa*	Pneumonia	Blood, sputum, pleural fluid	Tube thoracostomy	Nonsurvivor
19	*E. coli*	Renal abscess	Blood, abscess, urine	PCD	Survivor
20	*Salmonella* group B	Soft tissue abscess	Blood, abscess	Incision and drainage	Survivor

PCD: Percutaneous catheter drainage; TVR: Tricuspid valve replacement; VATS: Video-assisted thoracoscopic surgery
